# Africa-wide evaluation of host biomarkers in QuantiFERON supernatants for the diagnosis of pulmonary tuberculosis

**DOI:** 10.1038/s41598-018-20855-7

**Published:** 2018-02-08

**Authors:** Novel N. Chegou, Jayne S. Sutherland, Anna-Ritah Namuganga, Paul LAM Corstjens, Annemieke Geluk, Gebremedhin Gebremichael, Joseph Mendy, Stephanus Malherbe, Kim Stanley, Gian D. van der Spuy, Magdalena Kriel, Andre G. Loxton, Belinda Kriel, Felanji Simukonda, Yonas Bekele, Jacob A. Sheehama, Josefina Nelongo, Marieta van der Vyver, Atsbeha Gebrexabher, Habteyes Hailu, Maria M. Esterhuyse, Ida Rosenkrands, Claus Aagard, Martin Kidd, Desta Kassa, Adane Mihret, Rawleigh Howe, Jacqueline M. Cliff, Amelia C. Crampin, Harriet Mayanja-Kizza, Stefan H. E. Kaufmann, Hazel M. Dockrell, Tom H. M. Ottenhoff, Gerhard Walzl, AE-TBC consortium, Shirley P. McAnda, Olumuyiwa Owolabi, Abdou Sillah, Awa Gindeh, Simon Donkor, Toyin Togun, Martin Ota, Grace Muzanye, Mary Nsereko, Pierre Peters, Elisa M. Tjon Kon Fat, Claudia J. de Dood, Kees Franken, Jolien J. van der Ploeg-van Schip, Atsbeha Gebrezgeabher, Getnet Mesfin, Yohannes Belay, Yodit Alemayehu, Alemayehu Amberbir, Femia Chilongo, Rein Houben, Bamlak Tessema, Lawrence Yamuah, Faustina N. Amutenya, Lidia Monye, Scholastica Iipinge

**Affiliations:** 1 Present Address: DST/NRF Centre of Excellence for Biomedical Tuberculosis Research and SAMRC Centre for Tuberculosis Research, Division of Molecular Biology and Human Genetics, Faculty of Medicine and Health Sciences, Stellenbosch University, PO Box 241, Cape Town, 8000 South Africa; 2 Vaccines and Immunity, Medical Research Council Unit, Fajara, The Gambia; 3 Uganda-Case Western Research Collaboration - Makerere University, PO Box 663, Kampala, Uganda; 4 Department of Molecular Cell Biology, Leiden University Medical Centre, PO Box 9600, 2300 RC Leiden, The Netherlands; 5 Department of Infectious Diseases, Leiden University Medical Centre, PO Box 9600, 2300 RC Leiden, The Netherlands; 6 Ethiopian Public Health Institute, Addis Ababa, Ethiopia; 7 Karonga Prevention Study, Chilumba, Malawi; 8 Armauer Hansen Research Institute, Addis Ababa, Ethiopia; 9 School of Medicine, Faculty of Health Sciences, University of Namibia, Windhoek, Namibia; 10 Department of Immunology, Max Planck Institute for Infection Biology, Charitéplatz 1, 10117 Berlin, Germany; 11 Department of Infectious Disease Immunology, Statens Serum Institut, Copenhagen, 2300s Denmark; 12 Centre for Statistical Consultation, Department of Statistics and Actuarial Sciences, Stellenbosch University, Cape Town, South Africa; 13 Department of Immunology and Infection, London School of Hygiene and Tropical Medicine, Keppel Street, London, WC1E 7HT UK; 14 Present Address: URC Malawi Lab Project, PO Box 1921, Lilongwe, Malawi

## Abstract

We investigated host-derived biomarkers that were previously identified in QuantiFERON supernatants, in a large pan-African study. We recruited individuals presenting with symptoms of pulmonary TB at seven peripheral healthcare facilities in six African countries, prior to assessment for TB disease. We then evaluated the concentrations of 12 biomarkers in stored QuantiFERON supernatants using the Luminex platform. Based on laboratory, clinical and radiological findings and a pre-established algorithm, participants were classified as TB disease or other respiratory diseases(ORD). Of the 514 individuals included in the study, 179(34.8%) had TB disease, 274(51.5%) had ORD and 61(11.5%) had an uncertain diagnosis. A biosignature comprising unstimulated IFN-γ, MIP-1β, TGF-α and antigen-specific levels of TGF-α and VEGF, identified on a training sample set (n = 311), validated by diagnosing TB disease in the test set (n = 134) with an AUC of 0.81(95% CI, 0.76–0.86), corresponding to a sensitivity of 64.2%(95% CI, 49.7–76.5%) and specificity of 82.7%(95% CI, 72.4–89.9%). Host biomarkers detected in QuantiFERON supernatants can contribute to the diagnosis of active TB disease amongst people presenting with symptoms requiring investigation for TB disease, regardless of HIV status or ethnicity in Africa.

## Introduction

Tuberculosis (TB) remains a global health problem, with an estimated 10.4 million new TB patients and 1.4 million deaths due to TB, reported in 2015^1^. The most widely used diagnostic test for TB especially in resource-poor settings (sputum smear microscopy) has poor sensitivity, whereas the gold standard test (culture) has a long turnaround time (up to 42 days), is expensive, prone to contamination and requires extensive laboratory infrastructure^2^. The GeneXpert MTB/RIF test, arguably the most important recent advance in TB diagnostics, yields results within 2 hours and detects resistance to rifampicin as a proxy for MDR-TB diagnosis. However, despite the extensive roll out of the GeneXpert, cost effectiveness and the requirement for technical infrastructure limits its use in resource-poor settings. As a consequence, the test is mostly offered in centralized facilities with adequate laboratory infrastructure. In addition, an important limitation of tests based on sputum, including the GeneXpert, is their unsuitability for individuals with difficulty in providing sputum specimens and those with paucibacillary disease such as children^3^, HIV infected individuals and patients with extra-pulmonary TB^4^. Assays employing the detection of host inflammatory biosignatures may be beneficial, as they have shown promise for development as rapid point-of-care tests for both pulmonary and extrapulmonary TB^5–8^.

The tuberculin skin test (TST) and interferon gamma (IFN-γ) release assays (IGRAs) remain the most widely used and recommended immunological tests for the diagnosis of *Mycobacterium tuberculosis (Mtb)* infection^9^. However, these assays are not very useful in the diagnosis of active TB disease^9,10^. Moreover, they can be falsely negative in advanced TB cases, most likely due to T cell anergy^5,11^. Several investigations have shown the diagnostic potential of host markers other than IFN-γ, which can be detected in QuantiFERON supernatants in diagnosing TB disease^12–15^. In addition, investigations into the discovery of alternative infection-phase dependent antigens other than those used in IGRAs (ESAT-6/CFP-10/TB7.7; present in the QuantiFERON In Tube assay), have been attempted^16–20^.

We have previously shown a 3-marker host biosignature consisting of epidermal growth factor (EGF), macrophage inflammatory protein (MIP)-1β and interleukin (IL)-1α, which resulted in the correct classification of 87% of adult pulmonary TB cases and 91% of household contacts after cross validation following QuantiFERON stimulation of whole blood^12^. However, this requires validation in a larger cohort of TB cases, including HIV positive and HIV negative subjects, from different geographical regions. Therefore, the aim of the present study was to validate the diagnostic potential of the 3-marker biosignature together with promising markers identified in other studies for the diagnosis of TB disease amongst individuals with symptoms suggestive of TB from six African countries (Ethiopia, Gambia, Malawi, Namibia, South Africa and Uganda), irrespective of HIV co-infection.

## Results

### Study subjects

**Figure 1:**
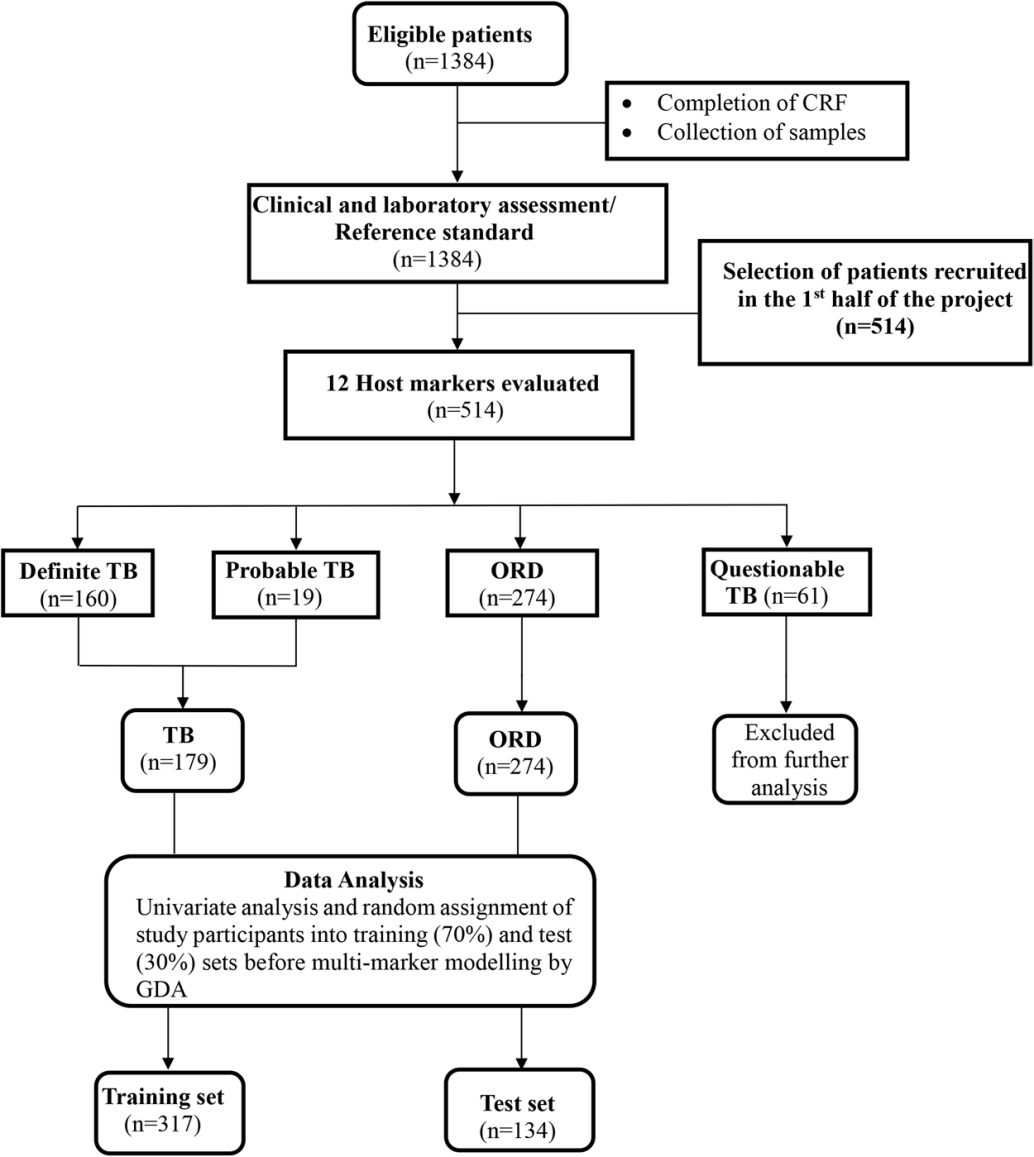
STARD flow diagram showing the study design and classification of study participants. CRF, case report form; TB, Pulmonary tuberculosis; ORD, Individuals presenting with symptoms and investigated for pulmonary TB but in whom TB disease was ruled out; ROC, Receiver operator characteristics; GDA, General discriminant analysis.

Samples from 514 study participants were evaluated (Fig. 1). Using the pre-established TB classification algorithm described in^21^, 160 (31.1%) of the study participants were definite pulmonary TB patients, 19 (3.7%) were probable TB patients, 274 (53.5%) were individuals with other respiratory diseases (ORDs), whereas 61(11.9%) of study participants had questionable disease status (Table 1). There was no significant difference between the proportion of patients with TB disease or ORD that were HIV infected (Chi-Square, p = 0.08) or between males and females with TB or ORD across sites (Chi-square, p = 0.16). The proportion of individuals presenting with symptoms requiring investigation for TB and who were finally diagnosed with TB disease was not the same across the different study sites (Table 1, Chi-square p < 0.01), with the TB patients slightly younger than those with ORD (35.7 ± 12.6 Vs. 36.8 ± 13.2; Chi-square p < 0.01, Table 1).

**Table 1:** Clinical and demographic characteristics of study participants.

Study site	SUN	AHRI	KPS	MRCG	UCRC	EHNRI	UNAM	Total
Participants (n)	75	76	76	72	67	73	75	514
Age, mean ± SD	38.6 ± 10.7	30.9 ± 12.6	38.6 ± 14.7	35.1 ± 13.4	32.5 ± 10.6	37.5 ± 14.1	36.2 ± 9.5	35.7 ± 12.6
Males, n(%)	35(47)	40(53)	39(51)	55(76)	42(63)	33(45)	39(52)	283(55)
HIV pos, n(%)	11(15)	11(14)	37(49)	0(0)	1(1)	18(25)	44(59)	122(24)
QFT pos, n(%)	48(66)	46(61)	25(33)	38(53)	54(81)	36(49)	40(53)	287(56)
^*^Definite, n(%)	20(27)	30(39)	6(8)	34(47)	24(36)	18(25)	28(37)	160(31)
^$^Probable, n(%)	0(0)	2(3)	5(7)	0(0)	2(3)	1(1)	9(12)	19(4)
^£^ORD, n (%)	46(61)	44 (58)	55(72)	35(49)	38(57)	39(53)	17(23)	274(53.3)
Questionable, n(%)	9(12)	0(0)	10(13)	3(4)	3(4)	15(21)	21(28)	61(11.9)
TB cases^♯^ (n)	20	32	11	34	26	19	37	179(34.2)
Age, mean ± SD	38.2 ± 9.2	28.7 ± 13.6	34.5 ± 13.2	31.5 ± 11.7	33.1 ± 9.5	27.9 ± 9.7	34.6 ± 9.5	35.7 ± 12.6
Males, n(%)	8(40)	16(50)	5(45)	30(88)	16(62)	11(58)	20(54)	106(59)
HIV pos, n(%)	4(20)	7(22)	4(36)	0(0)	1(4)	5(26)	23(62)	44(25)
QFT pos, n(%)	14(74)	26(81)	7(64)	26(76)	25(96)	12(63)	23(62)	133(75)
QFT neg, n(%)	4(21)	6(19)	4(36)	8(24)	1(4)	6(32)	11(30)	40(22)
QFT Indet., n(%)	1(5)	0(0)	0(0)	0(0)	0(0)	1(5)	3(8)	5(3)
ORD	45	44	55	35	38	39	17	274(53.3)
Age, mean ± SD	38.2 ± 11.2	32.4 ± 11.7	39.4 ± 15.5	39.3 ± 14.3	31.7 ± 11.0	38.6 ± 13.5	37.5 ± 10.6	36.8 ± 13.2
Males, n(%)	21(47)	24(55)	30(55)	22(63)	24(63)	14(36)	6(35)	141(51)
HIV pos, n(%)	6(13)	4(9)	26(47)	0(0)	0(0)	6(15)	9(53)	51(19)
QFT pos, n(%)	28(62)	20(45)	16(29)	11(31)	26(68)	20(51)	10(59)	131(48)
QFT neg, n(%)	17(3)	22(50)	33(60)	24(69)	11(29)	19(49)	7(41)	133(49)
QFT Indet., n(%)	0(0)	2(5)	6(11)	0(0)	1(3)	0(0)	0(0)	9(3)

*As previously described in^21^, participants were classified as Definite TB patients if MTB was isolated from their sputum samples by culture and/or when they had two positive smears and their symptoms responded well to treatment and/or if they had a single positive smear (only one smear done) and their chest X-rays were suggestive of pulmonary TB. ^$^Participants were classified as probable TB if they had one positive sputum smear (in the absence of a culture result) and their symptoms responded well to TB treatment, or when they had very suggestive chest X-rays and their symptoms responded to TB treatment. ^£^Participants were classified as ORD if they had negative cultures, negative smears, and negative chest X-rays and treatment was never initiated by healthcare providers. QuantiFERON results (positive, negative or indeterminate) were obtained using the software provided by the manufacturer. Abbreviations: SUN = Stellenbosch University, South Africa; AHRI = Armauer Hansen Research Institute, Ethiopia; KPS = Karonga Prevention Study, Malawi, MRC = Medical Research Council Unit, The Gambia, UCRC = Makerere University, Uganda; EHNRI = Ethiopian Public Health Institute, Ethiopia; UNAM = University of Namibia, Namibia; SD = standard deviation; QFT = QuantiFERON TB Gold In Tube; pos = positive; neg = negative; indet = indeterminate. ^♯^TB cases = Definite TB + probable TB cases.

### Diagnostic potential of individual host markers for TB disease

We compared all individuals diagnosed with TB disease (definite and probable TB; n = 179) to those with ORD (n = 274) for both unstimulated (N) and TB antigen induced values (Ag-N) responses for each individual marker; obtained by subtraction of the unstimulated (nil) from the antigen stimulated (Ag) responses, regardless of HIV infection status. These values were considered separate variables during statistical analysis in order to evaluate the contribution of each to diagnostic biosignatures.

Significant differences were observed for the unstimulated and/or antigen specific levels of eight markers (Table 2). The median unstimulated levels of VEGF, IFN-γ, sCD40L, TGF-α, IFN-α2 and MMP-9, and antigen-specific levels of IL-1ra, IFN-γ, TGF-α and MMP-9 were significantly higher in the TB patients whereas the unstimulated levels of MIP-1β and IL-1α were significantly higher in individuals with ORD (Table 2).

**Table 2:** Median levels of individual analytes (pg/ml) in TB patients (n = 179) and individuals with ORD (n = 274) and accuracies in the diagnosis of TB disease.

Analyte	TB (IQR)	ORD (IQR)	P-value	AUC (95% CI)	Cut-off	Sens (95% CI)	Spec (95% CI)
IL-1ra_Ag-N_	124.1(26.4–486)	78.7(0–361.1)	0.032	0.58 (0.51–0.65)	>90.3	0.60 (0.50–0.69)	0.52(0.43–0.60)
VEGF_N_	102.2(0.0–213)	40.7(0.0–118.5)	<0.001	0.63(0.58–0.68)	>74.8	0.60 (0.53–0.68)	0.60(0.54–0.65)
IFN-γ_N_	17.6(7.7–36)	5.6(1.1–14.0)	<0.001	0.72(0.67–0.77)	>8.7	0.73(0.66–0.78)	0.63(0.57–0.68)
IFN-γ_Ag-N_	115.8(21.5–340)	23.1(0.8–169.3)	<0.001	0.64(0.59–0.69)	>46.5	0.67 (0.60–0.74)	0.60 (0.54–0.66)
IFN-α2_N_	35.9(14.6–55)	20.0(7.7–39.6)	0.001	0.62(0.55–0.69)	>24.9	0.62(0.52–0.71)	0.58(0.49–0.66)
sCD40L_N_	2322.8(1197.4–4507)	1344.4(765.6–2611.2)	<0.01	0.63(0.58–0.69)	>1717	0.63(0.55–0.70)	0.60(0.54–0.66)
MIP-1β_N_	752.6(386.8–1673)	1289.1(651.2–2466.0)	<0.01	0.63(0.58–0.68)	<1164	0.62(0.54–0.69)	0.54 (0.49–0.61)
MIP-1β_A-N_	467.5(19.6–1160)	627.9(22.6–2013.8)	0.05	0.55(0.50–0.60)	<705.6	0.63(0.55–0.69)	0.49(0.43–0.55)
IL-1α_N_	9.1(0.5–29)	14.2(3.1–52.5)	0.017	0.50(0.45–0.56)	<4.8	0.55(0.47–0.62)	0.51(0.44–0.56)
TGF-α_N_	18.0(11.2–30)	9.8(4.2–19.1)	<0.001	0.68(0.63–0.73)	>12.9	0.71(0.64–0.78)	0.60(0.54–0.66)
TGF-α_A-N_	0.9(−1.8–6.0)	0.0(−1.8–2.9)	0.021	0.56(0.51–0.62)	>0.6	0.53(0.456–0.60)	0.60(0.54–0.66)
MMP-9_N_	809798.8(455045.7–1275200)	508208.0(298946.4–865698.0)	<0.001	0.66(0.60–0.71)	>618289	0.60(0.52–0.68)	0.61(0.55–0.67)
MMP-9_Ag-N_	36703.4(−40408–234300)	4119.5(−82800–113700)	0.005	0.58(0.52–0.64	>3578	0.59(0.50–0.67)	0.50(0.44–0.56)

Only analytes with p-values < 0.05 in the Mann Whitney U test are shown. IQR = Inter-quartile range, AUC = area under the ROC curve, Sens = sensitivity, spec = specificity N = Unistimulated levels, Ag-N = antigen specific levels.

When analysis was performed only in HIV uninfected individuals (135 TB Vs. 223 ORD), significant differences were observed for unstimulated levels of IL-1ra, VEGF, IFN-γ, IFN-α2, sCD40L, MIP-1β, TGF-α, MMP-2, MMP-9, and antigen-specific levels of IL-1ra, VEGF, IFN-γ, TGF-α and MMP-9 (see Supplementary Table S1). When only the definite TB cases (n = 160) were compared to all the ORDs, regardless of HIV infection status, significant differences were observed for the unstimulated levels of VEGF, IFN-γ, IFN-α2, sCD40L, MIP-1β, TGF-α, MMP-9 and the antigen-specific levels of TGF-α, IFN-γ, VEGF and IL-1ra (see Supplementary Table S2).

When the diagnostic accuracies of individual host markers were ascertained by ROC curve analysis, unstimulated IFN-γ was the only marker that showed diagnostic potential, with area under the ROC curve (AUC) of 0.72 (95% CI, 0.67–0.78) in all study participants, regardless of HIV status (Table 2), and also in HIV negative individuals only (AUC = 0.73; 95% CI, 0.68–0.78) (see Supplementary Table S1). When only the definite TB patients were compared to ORD, the AUCs for individual analytes ranged from 0.52 (95% CI, 0.45–0.60) for unstimulated IL-1ra, to 0.75(95% CI, 0.70–0.80) for unstimulated IFN-γ (see Supplementary Table S2), thereby confirming the limited utility of individual analytes, and also suggesting that *Mtb* antigen stimulation might not be necessary for optimal diagnosis of TB disease.

### Utility of the previous 3-marker EGF, MIP-1β and IL-1α signature in the diagnosis of TB disease

We evaluated the accuracy of the 3-marker (EGF, MIP-1β and IL-1α) biosignature identified in our previous small, case-control study^12^ in the present study. When evaluated on all study participants (regardless of HIV infection status), the 3-marker signature only diagnosed TB disease in the training sample set (70% of all study participants), with a sensitivity of 68.9% and specificity of 52.9%. In the test set (the remaining 30% of study participants) the sensitivity and specificity of the 3-marker model were 60.4% and 58.0% respectively. When evaluated in HIV negative participants only, the sensitivity and specificity of the 3-marker model (54.8% and 59.2% respectively) were equally poor in the test sample set, with similar results (although with higher specificity) obtained in the HIV positive individuals (sensitivity of 40% and specificity of 90% in the test set).

### Diagnostic Potential of other multi-marker biosignatures for TB disease

We next evaluated the predictive abilities of combinations between different host markers using general discriminant analysis (GDA). As not all host markers were evaluated at all study sites (IL-1ra and IFN-α2 were not evaluated on KPS, MRCG, and UCRC samples and TNF-α was not evaluated on MRCG samples), the GDA was performed twice: -we first evaluated diagnostic biosignatures in patients for whom all host markers had been evaluated. Secondly, we evaluated combinations between host markers that had been evaluated at all study sites (i.e. excluding IL-1ra, IFN-α2 and TNF-α), regardless of HIV infection status, or study site. Optimal prediction of TB disease Vs. ORD was achieved when markers were used in combinations of four.

When all host markers were analysed for available sites (n = 251), a four-marker signature comprising unstimulated IFN-γ, unstimulated TGF-α and antigen-specific levels of IL-1ra and MIP-1β diagnosed TB disease with a sensitivity of 70.7% (95% CI, 58.9–80.3%), and specificity of 81.2% (95% CI, 71.9–88.0%) in the training set (n = 176; 75 TB and 101 ORDs), and a sensitivity of 68.8% (95% CI, 50.0–83.3%) and specificity of 76.7% (95% CI, 61.0–87.7%) in the test sample set (n = 75; n = 32 TB and n = 43 ORD). The positive and negative predictive values of the biosignature in the test set were 68.8% (95% CI, 50.0–83.3%) and 76.7% (95% CI, 61.0–87.7%), respectively.

**Figure 2:**
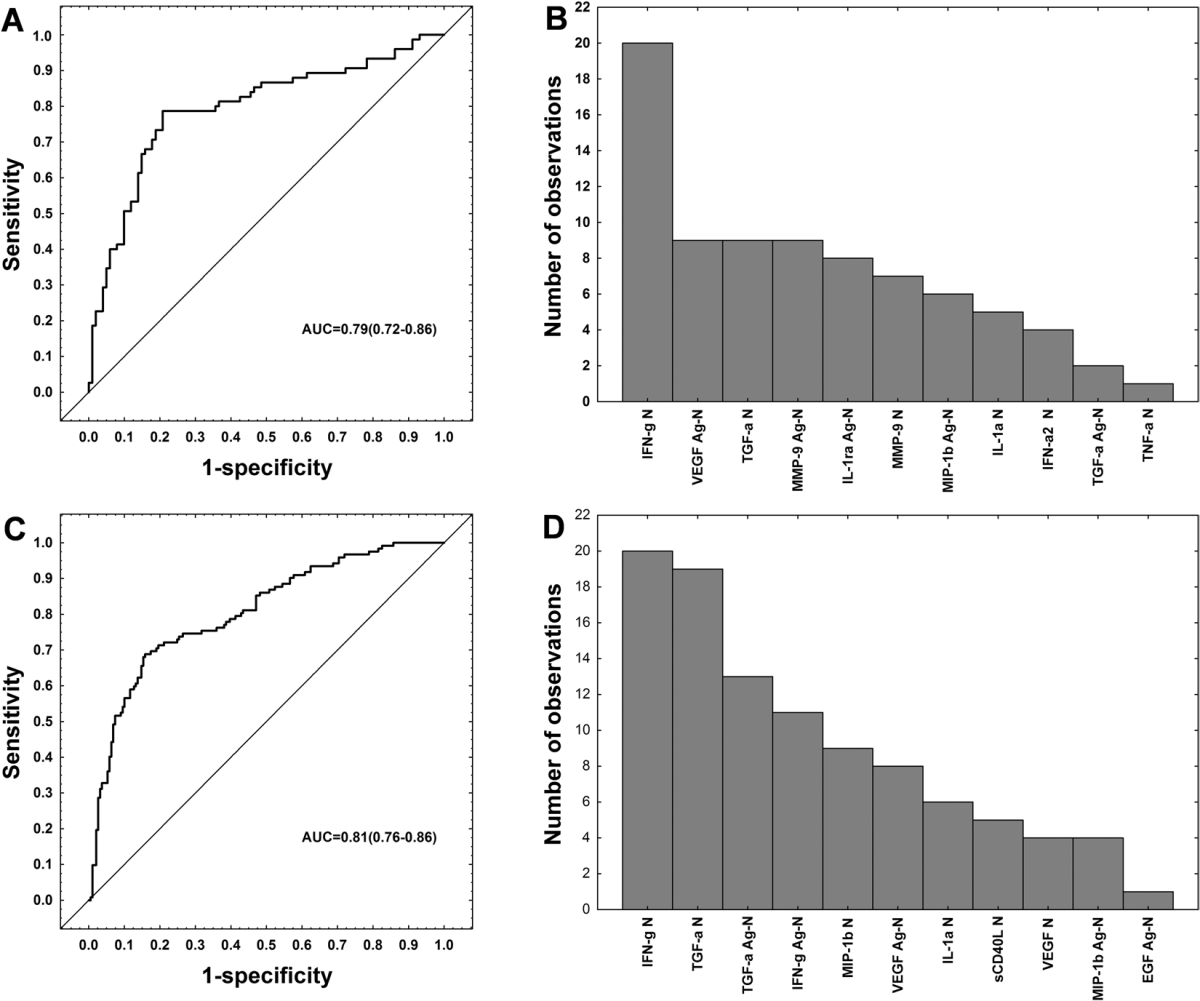
Accuracy of multi-marker models in the diagnosis of TB disease. Receiver operator characteristics (ROC) curve showing the accuracy of the most accurate four-marker biosignature (IFN-γ_nil_ + TGF-α_nil_ + IL-1ra_Ag-nil_ + MIP-1β_Ag-nil_) in the diagnosis of TB disease regardless of HIV infection status when all host markers evaluated were considered (251 study participants) (**A**), frequency of analytes in the top 20 general discriminant analysis (GDA) models that most accurately classified study participants as TB disease or ORD irrespective of HIV status when all host markers evaluated were considered (**B**), ROC curve showing the accuracy of the most accurate five-marker biosignature (IFN-γ_nil_ + MIP-1β_nil_ + TGF-α_nil_ + TGF-α_Ag-nil_ + VEGF_Ag-nil_) in the diagnosis of TB disease regardless of HIV status when analysis was done only on the host markers that were evaluated on all study participants (i.e., excluding IL-1ra, IFN-α2 and TNF-α) (**C**), and frequency of analytes in the top 20 GDA models that most accurately diagnosed TB disease regardless of HIV status when analysis was done only on the host markers that were evaluated on all study participants (**D**). The bar graphs (**B**,**D**) indicate the frequency of analytes in the most accurate GDA models.

When analysis was performed only on the host markers that were evaluated in all study participants, optimal prediction of TB disease was achieved when markers were used in combinations of five. The most accurate five-marker biosignature, comprised unstimulated levels of IFN-γ, MIP-1β and TGF-α and antigen-specific levels of TGF-α and VEGF, and diagnosed TB disease in the training sample set (n = 311; n = 122 TB, n = 189 ORD) with sensitivity of 68.9% (95% CI, 59.7–76.8%) and specificity of 83.1% (95% CI, 76.8–88.0%), and a sensitivity of 64.2% (95% CI, 49.7–76.5%) and specificity of 82.7% (95% CI, 72.4–89.9%) in the test set (n = 134; n = 53 TB and n = 81 ORD). The positive and negative predictive values of the five-marker biosignature in the test set were 70.8% (95% CI, 55.7–82.6%) and 77.9% (95% CI, 67.4–85.9%), respectively (Fig. 2, Table 3).

**Table 3:** Summary of diagnosticc biosignatures identified in this study.

**B**iosignature(i) obtained when all host markers (n = 12) were considered; implying limited numbers of study participants (N = 251), from 4 sites only, regardless of HIV status						
	Training set (N = 176, n = 75 TB, n = 101 ORD)		Test set (n = 75, n = 32TB; n = 43ORD)			
**Biosignature (i)** ^**a**^	**Sensitivity**	**Specificity**	**Sensitivity**	**Specificity**	**PPV**	**NPV**
IFN-γ_N_, TGF-α_N_, IL-_1RaAg-N_, MIP-1β_Ag-N_	70.7% (58.9–80.3)	81.2% (71.9–88.0)	68.8%(50.0–83.3)	76.7%(61.0–87.7)	68.8%(50.0–83.3)	76.7%(61.0–87.7)
Biosignature (ii) obtained when all study participants (n = 445*, all sites) were considered, implying limited numbers of markers (n = 9)						
Biosignature (ii)	Training set (n = 311; n = 122 TB, n = 189 ORD)		Test set (N = 134; n = 53 TB; n = 81 ORD)			
IFN-γ_N_, MIP-1β_N_, TGF-α_N_, TGF-_αAg-N_, VEGF_Ag-N_	68.9%(59.7–76.5)	83.1%(76.8–88.0)	^c^64.2%(49.7–76.5)	^d^82.7%(72.4–89.9)	70.8%(55.7–82.6)	77.9%(67.4–85.9)
**Biosignature (iii) obtained when all host markers (n = 12) were considered in HIV negative study participants only; implying limited numbers of study participants (N = 189), from 4 study sites**						
Biosignature (iii)	Training set: N = 133; n = 48 TB, n = 85 ORD		Test set: N = 56; n = 20 TB, n = 36 ORD			
IFN-γ_N_, IFN-α_N_, sCD40L_N_, IL-1α_N_, MMP-2_N_, MMP-9_N_, IFN-α2_Ag-N_	81.2%(66.9–90.6)	81.2%(70.9–88.5)	50.0%(27.9–72.1)	83.3%(66.5–93.0)	62.5%(35.9–83.7)	75.0(58.8–86.8)
Biosignature (iv) obtained when all HIV negative participants (n = 352*, all sites) were considered, implying limited numbers of markers (n = 9)						
Biosignature (iv)	Training set (N = 247; n = 92 TB, n = 155 ORD)		Test set (n = 105; n = 39 TB, n = 66 ORD)			
IFN-γ_N_, TGF-α_N_, IL-1α_N_, MMP-2_N_, EGF_Ag-N_, VEGF_Ag-N_, TGF-α _Ag-N_	73.9%(63.5–82.3)	84.5%(77.6–89.6)	51.3%(35.0–67.3)	77.3%(65.0–86.3)	57.1%(39.5–73.2)	72.9%(60.7–82.4)

All host markers were not evaluated at all study sites: IL-1ra and IFN-α2 were not evaluated on KPS, MRCG, and UCRC samples, whereas TNF-α was additionally not evaluated on MRCG samples for technical reasons. The GDA modelling procedure was therefore performed twice.

*Individuals with questionable TB status (see Table 1) were excluded.

^a^The sensitivity of biosignature (i) increased to 81.3% and specificity decreased to 56.0% when the biosignature was optimized for high sensitivity. ^b^The sensitivity of biosignature (ii) increased to77.4% and specificity decreased to 60.5% when the biosignature was optimized for high sensitivity. N = Unstimulated (nil) value, Ag-N = antigen-specific response obtained after subtraction of nil from antigen-stimulated value.

After optimization of biosignatures by selection of cut-off levels that would yield the best possible combinations of sensitivity and specificity, for possible future development of a screening test, the sensitivity of the four-marker biosignature in the test set increased to 81.3%, at the expense of lower specificity (56.0%, AUC = 0.79, 95%CI, 0.72–0.86), whereas the sensitivity of the five-marker model on all study participants (limited analytes) increased slightly to 77.4% with the specificity reducing to 60.5%, in the test set (AUC = 0.81, 95%CI, 0.76–0.86).

### Accuracy of biosignatures in HIV negative subjects

**Figure 3:**
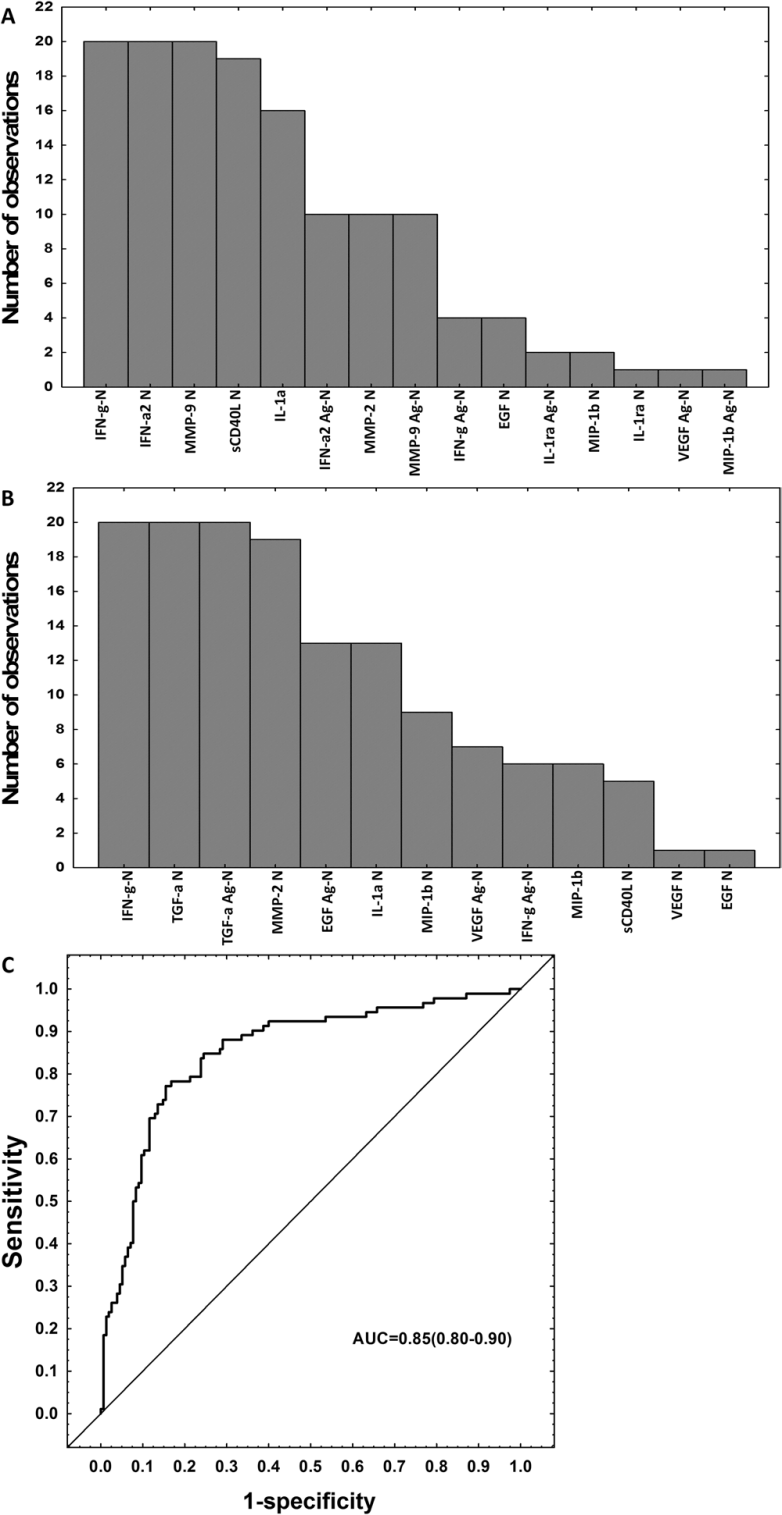
Accuracy of multi-marker models in the diagnosis of TB disease in the absence of HIV infection. Frequency of analytes in the top 20 general discriminant analysis (GDA) models that most accurately classified study participants regardless of HIV infection status, when all host markers evaluated were considered (limited numbers of study participants) (**A**), frequency of analytes in the top 20 GDA models that most accurately classified study participants as TB disease or ORD irrespective of HIV status when all study participants (limited numbers of host markers) were considered (**B**), ROC curve showing the accuracy of the most accurate seven-marker biosignature (IFN-γ_N_, TGF-α_N_, IL-1α_N_, MMP-2_N_, EGF_Ag-N_, VEGF_Ag-N_, TGF-α _Ag-N_) in the diagnosis of TB disease regardless of HIV status when analysis was done only on the host markers that were evaluated on all study participants (i.e., excluding IL-1ra, IFN-α2 and TNF-α) (**C**). The bar graphs (**A**,**B**) indicate the frequency of analytes in the top 20 most accurate GDA models.

To investigate whether the accuracy of the biomarker combinations was influenced by HIV infection, we stratified the study participants according to HIV status and repeated the GDA in the HIV uninfected participants. For analysis of all analytes where available, a seven-marker biosignature comprising unstimulated levels of IFN-γ, IFN-α2, sCD40L, IL-1α, MMP-2, MMP-9 and antigen-specific IFN-α2 diagnosed TB disease with a sensitivity of 81.2% (95% CI 66.9–90.6%) and specificity of 81.2% (95% CI, 70.9–88.5%) in the training sample set (n = 133; n = 48 TB, n = 85 ORD), and a sensitivity of 50.0% (95% CI 27.9–72.1%), and specificity of 83.3% (95% CI 66.5–93.0%) in the test set (n = 56; n = 20 TB and n = 36 ORD). The positive and negative predictive values of the biosignature in the test set were 62.5% (95% CI, 35.9–83.7%) and 75.0% (95% CI, 58.5–86.8%) respectively. When the analysis was done only on the host markers that were evaluated at all study sites, a seven-marker combination comprising unstimulated levels of IFN-γ, TGF-α, IL-1a, MMP-2 and the antigen-specific levels of EGF, VEGF and TGF-α diagnosed TB disease with a sensitivity of 73.9% (95%CI, 63.5–82.3%) and specificity of 84.5% (95% CI 77.6–89.6%) in the training set (n = 247; n = 92TB, n = 155 ORD), and a sensitivity of 51.3% (95% CI 35.0–67.3% and specificity of 77.3% (95% CI, 65.0–86.3%) in the test set (n = 105; n = 39 TB, n = 66 ORD). The positive and negative predictive values of the biosignature in the test set were 57.1% (95% CI, 39.5–73.2%) and 72.9% (95% CI, 60.7–82.4%), respectively. Attempts to optimize the biosignature by selection of better cut-off values resulted in a sensitivity and specificity of both 67% in the test sample set. The frequency of host markers in the most accurate prediction models for the diagnosis of TB in the absence of HIV infection is shown in Fig. 3.

### Utility of bisignatures in the diagnosis of smear positive and smear negative TB

Given that smear microscopy remains the most widely used test in resource poor settings, we evaluated the utility of the biosignatures identified in this study (Table 3) in the diagnosis of TB disease in smear positive and smear negative TB patients, regardless of the HIV infection status of study participants. The AUCs for all the four different biosignatures ranged from 0.81 (95% CI, 0.73–0.90) to 0.83 (95% CI, 0.77–0.88) for the diagnosis of smear positive TB disease, and from 0.70 (95% CI, 0.60–0.79) to 0.74 (95% CI, 0.63–0.84) for the diagnosis of smear negative TB (Table 4). The most accurate biosignature for the diagnosis of smear positive TB (IFN-γ_N_, TGF-α_N_, IL-1α_N_, MMP-2_N_, EGF_Ag-N_, VEGF_Ag-N_ and TGF-α _Ag-N_) performed in this study group with a sensitivity of 75.7% (28/37) and specificity of 80% (52/65) in the test set, whereas the most accurate biosignature for the diagnosis of smear negative TB (IFN-γ_N_, IFN-α_N_, sCD40L_N_, IL-1α_N_, MMP-2_N_, MMP-9_N_ and IFN-α2_Ag-N_) performed with a sensitivity of 60% (24/40) and specificity of 70.8% (102/144).

**Table 4:** Performance of host biosignatures in smear positive and smear negative TB patients regardless of HIV infection status.

Biosignature	Accuracy measure	Accuracy in smear Positive TB patients		Accuracy in Smear Negative TB Patients	
		Training set	Test set	Training set	Test set
Biosignature (i)	AUC		0.82 (0.75–0.90)		0.70 (0.61–0.79)
IFN-γ_N_, TGF-α_N_, IL-_1RaAg-N_, MIP-1β_Ag-N_	Sensitivity	72.3% (34/47)	80.0% (16/20)	60% (24/40)	57.5% (23/40)
	Specificity	85.1% (86/101)	79.5% (35/44)	73.1% (106/145)	71.0% (103/145)
Biosignature (ii)	AUC		0.82 (0.77–0.88)		0.70 (0.60–0.79)
IFN-γ_N_, MIP-1β_N_, TGF-α_N_, TGF-_αAg-N_, VEGF_Ag-N_	Sensitivity	71.8% (61/85)	56.8% (21/37)	57.8% (26/45)	55.6% (25/45)
	Specificity	83.7% (128/153)	90.8% (59/65)	75.2% (164/218)	72.9% (159/218)
Biosignature (iii)	AUC		0.81 (0.73–0.90)		0.74 (0.63–0.84)
IFN-γ_N_, IFN-α_N_, sCD40L_N_, IL-1α_N_, MMP-2_N_, MMP-9_N_, IFN-α2_Ag-N_	Sensitivity	73.9% (34/46)	75.0% (15/20)	65% (26/40)	60% (24/40)
	Specificity	82.2% (83/101)	90.7% (39/43)	72.9% (105/144)	70.8% (102/144)
Biosignature (iv)	AUC		0.83 (0.77–0.88)		0.70 (0.60–0.79)
IFN-γ_N_, TGF-α_N_, IL-1α_N_, MMP-2_N_, EGF_Ag-N_, VEGF_Ag-N_, TGF-α _Ag-N_	Sensitivity	70.6% (60/85)	75.7% (28/37)	57.8% (26/45)	55.6% (25/45)
	Specificity	85.0% (130/153)	80.0% (52/65)	75.2% (164/218)	72.9% (159/218)

Cut-off values for determination of sensitivity and specificity were selected based on the Younden’s Index.

## Discussion

The development of rapid, accurate, inexpensive and sputum independent TB diagnostic tools, that are suitable for use in high burden but resource-constrained settings, remains an important priority in the fight against TB^22^. Ideally, such tools should yield results within 20 minutes^22^ and based on easily obtainable samples such as serum, urine or other *ex vivo* bodily fluids^8^. In the absence of such tools, overnight antigen-stimulated tests, employing simple detection platforms such as lateral flow technology might be beneficial, provided that they are accurate, inexpensive and require minimal infrastructure^23^. In the present study, we evaluated the usefulness of host biomarkers detected in overnight whole blood culture (QuantiFERON) supernatants as potential diagnostic markers for TB disease in a large, multi-center trial, involving seven field sites, situated in six African countries. The concentrations of IL-1ra, IFN-α2, VEGF, sCD40L, MIP-1β, MMP-9, TGF-α and IFN-γ with or without antigen (ESAT/CFP-10/TB7.7) stimulation discriminated between TB patients and individuals with ORD. Although unstimulated IFN-γ levels showed promise, no single marker was sufficiently discriminatory as a stand-alone diagnostic marker for active TB. A previous 3-marker signature (EGF, MIP-1β and IL-1α)^12^ did not validate in the present multi-site study. However, four- and five-marker biosignatures comprising unstimulated levels of IFN-γ, MIP-1β and TGF-α, and the antigen-specific levels of TGF-α, VEGF, IL-1ra and MIP-1β diagnosed TB disease with sensitivity up to 81.3% and specificity up to 82.7%, regardless of HIV infection status, depending on which biomarkers were used in combination but specificity was generally low when biosignatures were optimized for high sensitivity. The most accurate four-marker biosignature comprising of the unistimulated levels of IFN-γ and TGF-α and the antigen specific levels of IL-1Ra and MIP-1β diagnosed TB disease in all study participants with a sensitivity of 81.3% and a specificity of 56.0%.

Although there are many stages in the *Mtb* infection/disease spectrum^24^, rapid discrimination between individuals who are infected with *Mtb* as evidenced by T cell reactivity to *Mtb* antigens, from those with active TB disease and for whom treatment is urgently needed, remains an important question. This is especially the case in the usually resource-constrained but high burden settings, with high prevalences of LTBI and poor diagnostic facilities. The development of IGRAs remains the most important recent advance in the development of immunodiagnostic tests for TB. However, it is widely known that IGRAs do not discriminate between active TB disease and LTBI, and IGRA responses have a poor predictive value as they do not correlate with any of the stages in the *Mtb* infection/disease spectrum^9^. In an attempt to identify alternative T-cell based diagnostic approaches, different researchers have investigated several alternative antigens to ESAT-6/CFP-10^16,25,26^ and more relevant to the current study, alternative host markers other than IFN-γ, detected after stimulation with ESAT-6/CFP-10/TB7.7 in QuantiFERON tubes, as reviewed in^18^. While different host biomarker signatures have been shown to be potentially useful, most of the work reported so far has been in small, single-site case-control studies and mostly in HIV uninfected individuals. Comparisons between these different, relatively small studies, is difficult, due to the high variability in both the types of patients, and the host markers investigated. Given the heterogeneity in immune responses against *Mtb* antigens across different populations^18^, it is imperative that any promising immunological assays be evaluated in large, multi-site studies, to ascertain the possible global applicability of the findings. As far as we are aware, our study is the first attempt to accomplish this. We therefore aimed to further elucidate the diagnostic potential of the most promising host markers so far identified in QuantiFERON supernatants, in a relatively large, highly diverse Pan-African cohort of patients that presented with signs and symptoms requiring investigation for pulmonary TB. Our objective was to refine and validate these biosignatures, and to identify a biosignature that could potentially be incorporated into easy-to-perform test platforms such as lateral flow based systems, which were recently investigated in different African countries^8,23^.

Our finding that four- to five-marker biosignatures detected in QuantiFERON supernatants have potential in the diagnosis of TB disease regardless of HIV infection status in this relatively large, multi-center study, attests to the promising nature of possible future tests based on such biosignatures. Although the performance of these biosignatures might be sub-optimal when compared to recent serum and plasma-based studies^21,27^, a diagnostic test based on these biosignatures expectedly has value in the difficult to diagnose TB cases, especially if optimized for high sensitivity, to enable the use of such biosignatures as screening tools for active TB disease. The markers need to be incorporated into an inexpensive and easily performed test system applicable in resource constrained settings. Optimization of threshold values for the biosignatures, to accurately determine how the markers could be used in potential rule-in or rule-out tests is ongoing. However, biosignatures such as those investigated in the current study may not be useful alone, and may have to be combined with other clinical and laboratory parameters.

Regarding the possible use of the biosignatures in the field, the recently published FIND/WHO target product profiles (TPPs) recommend that a non-sputum based biomarker test performs with an overall sensitivity ≥80% in adults, an optimal sensitivity ≥98% in the diagnosis of smear positive/culture positive TB and ≥68% for smear negative/culture positive TB, and with an optimal specificity ≥98% against a microbiological reference standard^22^. The most accurate biosignatures identified in the current study; with a sensitivity of 75.7% and specificity of 80% in the diagnosis of smear positive TB, and a sensitivity of 60% and specificity of 70.8% in the diagnosis of smear negative TB in the test set, performed sub-optimally when considering the FIND/WHO TPPs. However, as observed when the biosignatures were evaluated in all study participants regardless of HIV infection status, selection of better cut-off values yielded the desired optimal sensitivities (≥98%) with all the different biosignatures, but this was usually at the expense of lower specificity (data not shown). More work is currently being done to further optimise these biosignatures, including the improvement of the signatures by addition of more recently identified promising host markers.

Recently we developed field-friendly assays utilizing the lateral flow (LF) format in combination with upconverting phosphor (UCP) technology that allows quantitative detection of multiple biomarkers directly in culture supernatants^28,29^. For field-friendly use, the concentrations of analytes could be determined using a hand-held LF strip scanner, allowing point-of-care screening in remote settings (where culture and GeneXpert facilities are absent). Prototypes of UCP-LF tests, are currently being investigated within our consortium^8,23^. In the context of the current study, the levels of the biomarkers may be measured directly in the Quantiferon tubes after stimulation with antigens using the lateral flow test, without the need for ELISAs as it is currently done in IGRAs. The importance of discriminating between TB and ORD when patients present with similar symptoms to primary healthcare facilities often without even X-ray facilities cannot be overstated. Considering all patients in this study had some form of respiratory infection resulting in non-specific inflammatory responses in the lungs, the fact that we could identify biosignatures with up to 83% specificity for TB holds promise for future development of rapid screening tests for TB^22^. It will be important to further investigate these signatures in difficult-to-diagnose TB cases such as childhood TB and extrapulmonary TB cases in future studies. Furthermore, as discussed in another study^21^, it will be important if future studies include individuals with firmly established alternative diagnoses as the ORD group in the current study was not worked-up for confirmation of alternative diseases as patients were recruited at primary healthcare settings where diagnostic tools to confirm such alternative diagnoses are lacking. As the study participants included in the present study were patients presenting at primary health care facilities with symptoms and then investigated for TB disease, individuals with HIV infection were not investigated further for the purposes of staging with CD4 counts and viral loads. Furthermore, data on antiretroviral therapy was not collected as our interest was in the performance of biomarkers in patients with TB symptoms, regardless of whether they were HIV infected or not. It is therefore not certain how severe HIV infection might influence the accuracies of the biosignatures and that needs to be investigated in future studies.

In conclusion, the current study demonstrates that host biosignatures including four or five analytes obtained from *Mtb*-antigen stimulated or non-stimulated, overnight whole blood culture supernatants may be useful for the diagnosis of TB disease, in patients presenting with symptoms that are similar to TB, regardless of HIV infection status or ethnicity in Africa. Implementation of host biosignatures in low-complexity screening tools, as our previously described UCP-LF assay platform for cyto-/chemokine detection, may be beneficial in situations where sputum-based microbiological tests are not feasible.

## Methods

### Study participants

We prospectively recruited adults who presented with symptoms requiring investigation for pulmonary TB disease at primary health care clinics at seven field sites, situated in six African countries. As previously reported^19,21^, the clinics served as field study sites for researchers at Stellenbosch University (SUN), South Africa; Makerere University (UCRC), Uganda; Medical Research Council Unit The Gambia (MRCG); Karonga Prevention Study (KPS), Malawi; the University of Namibia (UNAM), Namibia, Armaur Hansen Research Institute (AHRI), Ethiopia; the Ethiopian Public Health Institute (EHNRI), Ethiopia. Study participants were recruited between November 2010 and November 2012.

As described previously^8,19,21,27,30^, study participants presented with persistent cough lasting ≥2 weeks and at least one of either fever, malaise, recent weight loss, night sweats, knowledge of close contact with a confirmed TB patient, haemoptysis, chest pain or loss of appetite. Participants were eligible for the study if they were 18 years or older and willing to give written informed consent, including consent for HIV testing. Patients were excluded if they were pregnant, had not been residing in the study community for more than 3 months, were severely anaemic (haemoglobin <10 g/l), had concurrent malaria infection (The Gambia), were on anti-TB treatment, received anti-TB treatment in the previous 90 days or if they received quinolone or aminoglycoside antibiotics during the past 60 days. At enrolment, case report forms were completed for all study participants and sputum and blood samples collected for analysis as described below. Harmonized clinical and laboratory protocols were used across the study sites. All study procedures were conducted according to the Declaration of Helsinki. The study protocol was approved by the Health Research Ethics Committees of all the participating institutions.

### Sample collection and diagnostic tests

Whole blood was collected directly into QuantiFERON TB Gold In Tube tubes (1 ml per tube) as recommended by the manufacturer (Qiagen, Germany). The tubes were transported at ambient conditions to the respective laboratories and incubated overnight (18 to 22 hours) at 37 °C, 5% CO_2_. Tubes were then centrifuged at 2,500 g for 15 minutes and supernatants harvested, aliquoted and frozen (−80 °C) until use.

Sputum samples were cultured either using the MGIT method (BD Biosciences) or on Lowenstein Jensen media, depending on the facilities at each site. Specimens demonstrating growth of microorganisms were examined for acid-fast bacilli using the Ziehl-Neelsen method, followed by either Capilia TB testing (TAUNS, Numazu, Japan) or standard biochemical methods, to confirm the isolation of organisms of the *Mtb* complex, before being designated as positive cultures.

### Classification of study participants and reference standard

As described previously^19,21,27^, study participants were classified as either definite TB patients, probable TB patients, participants with other respiratory diseases (ORD) or questionable disease status, using a combination of clinical, radiological, and laboratory findings. As previously reported^21,27^, individuals with ORDs had a range of other diagnoses, including upper and lower respiratory tract infections (viral and bacterial infections), and acute exacerbations of chronic obstructive pulmonary disease or asthma. However, attempts to identify any other organisms by bacterial or viral cultures were not made.

A total of 1,384 individuals (25.7% of whom were HIV infected) were enrolled at all sites during the study period. However, the current investigation only involved 514 individuals (24%, n = 122 HIV positive) that were recruited during the first half of the project, consistently using the same inclusion criteria. In assessing the accuracy of host biosignatures in the diagnosis of TB disease, all definite and probable TB cases were classified as “TB”, and then compared to participants with ORDs, whereas questionables were excluded from the main analysis (Fig. 1).

### Immunoassays

IFN-γ concentrations in QuantiFERON supernatants were determined using the QuantiFERON TB Gold ELISA kit and the results interpreted for *Mtb* infection using the analysis software provided by the manufacturer (Qiagen, Germany). QuantiFERON results were only used to determine the *Mtb* infection status of study participants and not for clinical management of patients.

The concentrations of the previously identified three markers (EGF, MIP-1β, IL-1α), as well as those of nine other markers that have shown potential including soluble CD40 ligand (sCD40L), transforming growth factor (TGF)-α, vascular endothelial growth factor (VEGF), IFN-α2, interleukin-1 receptor antagonist (IL-1ra), tumour necrosis factor (TNF)-α, IFN-γ, matrix metalloproteinase (MMP)-2 and MMP-9, were evaluated in aliquots of the unstimulated (nil) and *M.tb* antigen-stimulated QuantiFERON supernatants from each participant using customized Milliplex Luminex kits (Merck Millipore, Billerica, MA, USA), on the Bio Plex platform (Bio Rad Laboratories, Hercules, CA, USA). QuantiFERON mitogen control supernatants were not evaluated as they did not contribute to diagnostic biosignatures in previous studies^12^. All samples were evaluated blinded to the clinical classification and QuantiFERON status of study participants. The Bio Plex manager software, version 6.1 (Bio Rad Laboratories), was used for bead acquisition and analysis of median fluorescence intensity.

### Statistical analysis

Differences in the concentrations of host markers between the individuals with TB disease and those with ORDs were determined using the Mann–Whitney U test. The diagnostic accuracy for individual markers was ascertained by receiver operator characteristics (ROC) curve analysis. Optimal cut-off values and associated sensitivity and specificity were selected based on the Youden’s index^31^. The predictive abilities of combinations of analytes were investigated by performing best subsets general discriminant analysis (GDA)^31,32^, following the training/test set approach. Briefly, participants were randomly assigned into a training sample set (70%) or test set (30%), regardless of HIV infection status and biosignatures identified on the training set were validated on the test set. These training and test sets were selected using random sampling, stratified on the dependent (TB) variable. The data were analysed using Statistica (Statsoft, Ohio, USA) and Prism (Graph Pad Prism, San Diego, CA, USA).

### Availability of materials and data

The data presented in the current manuscript are available upon request from Gerhard Walzl or Novel N Chegou.

### Ethical approval and informed consent

All study procedures were conducted according to the Declaration of Helsinki. The study protocol was approved by the Health Research Ethics Committees of all the participating institutions.

## Electronic supplementary material

Supplementary Information
