# Supplementary material for: Africa-wide evaluation of host biomarkers in QuantiFERON supernatants for the diagnosis of pulmonary tuberculosis
Source: Sci Rep. 2018 Feb 8;8:2675. doi: 10.1038/s41598-018-20855-7 (PMC5805775; doi:10.1038/s41598-018-20855-7)
Supplement: Supplementary file 1 — Supplementary Information [file 41598_2018_20855_MOESM1_ESM.pdf]

Africa-wide evaluation of host biomarkers in QuantiFERON supernatants for the diagnosis of pulmonary tuberculosis:

Supplementary Results

Novel N. Chegou[1], Jayne S. Sutherland[2], Anna-Ritah Namuganga[3], Paul LAM Corstjens[4], Annemieke Geluk[5], Gebremedhin Gebremichael[6], Joseph Mendy[2], Stephanus Malherbe[1], Kim Stanley[1], Gian D. van der Spuy[1], Magdalena Kriel[1], Andre G. Loxton[1], Belinda Kriel[1], Felanji Simukonda[7,#], Yonas Bekele[8], Jacob A Sheehama[9], Josefina Nelongo[9], Marieta van der Vyver[9], Atsbeha Gebrexabher[6], Habteyes Hailu[6], Maria M. Esterhuyse[10], Ida Rosenkrands[11], Claus Aagard[11], Martin Kidd[12], Desta Kassa[5], Adane Mihret[8], Rawleigh Howe[8], Jacqueline M. Cliff[13], Amelia C. Crampin[7], Harriet Mayanja Kizza[2], Stefan H.E. Kaufmann[10], Hazel M Dockrell[13], Tom HM Ottenhoff[5], and Gerhard Walzl[*1] for the AE-TBC consortium

Affiliations:

[1]DST/NRF Centre of Excellence for Biomedical Tuberculosis Research and SAMRC Centre for Tuberculosis Research, Division of Molecular Biology and Human Genetics, Faculty of Medicine and Health Sciences, Stellenbosch University, PO Box 241, Cape Town, 8000, South Africa

[2]Vaccines and Immunity, Medical Research Council Unit, Fajara, The Gambia

[3]Uganda-Case Western Research Collaboration - Makerere University, PO Box 663, Kampala, Uganda

[4]Department of Molecular Cell Biology, Leiden University Medical Centre, PO Box 9600, 2300 RC Leiden, The Netherlands

[5]Department of Infectious Diseases, Leiden University Medical Centre, PO Box 9600, 2300 RC Leiden, The Netherlands

[6]Ethiopian Public Health Institute, Addis Ababa, Ethiopia

[7]Karonga Prevention Study, Chilumba, Malawi

[8]Armauer Hansen Research Institute, Addis Ababa, Ethiopia

[9]School of Medicine, Faculty of Health Sciences, University of Namibia, Namibia

[10]Department of Immunology, Max Planck Institute for Infection Biology, Charitéplatz 1, 10117 Berlin, Germany

[11]Department of Infectious Disease Immunology, Statens Serum Institut, Copenhagen 2300s, Denmark

[12]Centre for Statistical Consultation, Department of Statistics and Actuarial Sciences, Stellenbosch University, Cape Town, South Africa
[13]Department of Immunology and Infection, London School of Hygiene and Tropical Medicine, Keppel Street, London WC1E 7HT, UK
[#]Currently at URC Malawi Lab Project, PO Box 1921, Lilongwe, Malawi

***Corresponding Author:** Gerhard Walzl, DST/NRF Centre of Excellence for Biomedical Tuberculosis Research and SAMRC Centre for Tuberculosis Research, Division of Molecular Biology and Human Genetics, Faculty of Medicine and Health Sciences, Stellenbosch University, PO Box 241, Cape Town, 8000, South Africa. Telephone: +27 21 938 9124, E-mail: gwalzl@sun.ac.za

Alternate corresponding author: Dr Novel N Chegou, DST/NRF Centre of Excellence for Biomedical Tuberculosis Research and SAMRC Centre for Tuberculosis Research, Division of Molecular Biology and Human Genetics, Faculty of Medicine and Health Sciences, Stellenbosch University, PO Box 241, Cape Town, 8000, South Africa. Telephone: +27 21 938 9953, E-mail: novel@sun.ac.za

Supplementary Table 1: Median levels of individual analytes (pg/ml) in HIV uninfected study participants (n=135 TB Vs. n=223 ORD) and accuracies in the diagnosis of TB disease as determined by area under the ROC curve. Only analytes with p-values <0.05 in the Mann Whitney U test are shown. IQR= Inter-quartile range, AUC = area under the ROC curve, $_{N}$= Unistimulated levels, $_{Ag\text{-}N}$ = antigen specific levels, *The negative median values indicate that the value obtained in the antigen stimulated tube was lower than the value obtained in the nil tube and this phenomenon has been observed in previous studies[1].

Analyte	TB (IQR)	ORD (IQR)	P-value	AUC (95% CI)
**IL-1ra$_{N}$**	386.7(200.7-646.5)	295.5(159.2-525.4)	0.04	0.59(0.51-0.67)
**VEGF$_{N}$**	102.8(0.0-226.0)	55.4(0.0-119.0)	0.002	0.61(0.55-0.68)
**IFN-γ$_{N}$**	17.79(9.0-34.9)	6.1(1.9-15.30	<0.0001	0.73(0.68-0.78)
**IFN-α2$_{N}$**	36.2(17.0-53.4)	20.0(7.7-39.3)	0.001	0.64(0.56-0.72)
**sCD40L$_{N}$**	2625(1387-4815)	1434(835.8-2701)	<0.0001	0.67(0.61-0.73)
**MIP-1β$_{N}$**	1000(418.3-2132)	1324(684.9-2699)	0.003	0.59(0.53-0.66)
**TGF-α$_{N}$**	20.0(12.9-31.0)	10.2(4.2-19.1)	<0.0001	0.69(0.64-0.75)
**MMP-2$_{N}$**	119653(75661-152183)	123161(102812-164515)	0.042	0.56(0.50-0.63)
**MMP-9$_{N}$**	910512(516362-$1.344x10^{6}$)	540324 (301475-899608)	<0.0001	0.69(0.63-0.75)
**IL-1ra$_{Ag\text{-}N}$**	241.2(69.7-1002.0)	102.2(-10.7-400.2)	0.005	0.62(0.54-0.71)
***VEGF$_{Ag\text{-}N}$**	-20.9 (-102.5-0.0)	-0.0(-36.7-0.0)	0.006	0.59(0.52-0.65)
**IFN-γ$_{Ag\text{-}N}$**	151.4(29.8-438.7)	31.1(2.6-217.8)	<0.0001	0.64(0.58-0.70)
***TGF-α$_{Ag\text{-}N}$**	1.3(-1.9-7.9)	0.0(-1.6-3.1)	0.023	0.57(0.50-0.64)
***MMP-9$_{Ag\text{-}N}$**	75895(-36060-289983)	19103(-81433-121961)	0.005	0.60(0.53-0.66)

Supplementary Table 2: Median levels of individual analytes (pg/ml) obtained in the definite TB cases (n=160) and accuracies in the discriminating between definite TB and other respiratory diseases (n=274) regardless of HIV infection status. Only analytes with p-values <0.05 in the Mann Whitney U test are shown. IQR= Inter-quartile range, AUC = area under the ROC curve, $_N$= Unistimulated levels, $_{Ag-N}$ = antigen specific levels. *The negative median values indicate that the value obtained in the antigen stimulated tube was lower than the value obtained in the nil tube and this phenomenon has been observed in previous studies [1], #not significant; marker shown because it was one of the promosing markers identified in our previous small study[1].

ANALYTE	TB (IQR)	ORD (IQR)	P-VALUE	AUC (95% CI)
**VEGF$_N$**	102.2(0.0-221.4)	40.7(0.0-118.7)	< 0.0001	0,63 (0.58-0.69)
***VEGF$_{AG-N}$**	-25.29(-93.4-0.0)	0.0(-35.2-0.0)	0,0002	0.61(0.55-0.67
***IL-1RA$_{AG-N}$**	173.0(31.2-596)	78.7(-33.7.361.9)	0,0052	0.61(0.54-0.68)
**IFN-Γ$_N$**	18.7(9.0-38.3)	5.6(1.1-14.3)	<0.0001	0.75(0.71-0.80)
**IFN-Γ$_{AG-N}$**	140.3(27.6-353.8)	23.1(0.75-169.4)	<0.0001	0.67(0.62-0.73)
**IFN-A2$_N$**	35.2(14.0-52.3)	20.0(7.3-39.9)	0,0046	0.61(0.54-0.69)
**SCD40L$_N$**	2400(1217-4587)	1344(765.6-2611)	<0.0001	0.64(0.59-0.70)
**MIP-1B$_N$**	771.0(379.6-1737)	1289(648.5-2511)	<0.0001	0.62(0.57-0.68)
**TGF-A$_N$**	18.1(11.9-30.3)	9.8(4.2-19.1)	<0.0001	0.69(0.64-0.74)
***TGF-A$_{AG-N}$**	1.4(-1.4-7.3)	0.0(-1.8-2.9)	0,0011	0.59(0.54-0.66)
**MMP-9$_N$**	828920(491573-$1.284X10^{6}$)	508208(298606-868733)	<0.0001	0.68(0.62-0.73)
**MMP-9$_{AG-N}$**	44076(-40408-242490)	4119(-84693-113762	0,0030	0.59(0.53-0.66)
**#IL-1A$_N$**	12.5(1.2-29.7)	14.2(3.1-53.1)	0,0975	0.55(0.49-0.61)

References

1 Chegou, N. N., Black, G. F., Kidd, M., van Helden, P. D. & Walzl, G. Host markers in QuantiFERON supernatants differentiate active TB from latent TB infection: preliminary report. *BMC pulmonary medicine* **9**, 21, doi:10.1186/1471-2466-9-21 (2009).
